# Searching for Clues in the Diagnosis of McArdle Disease

**DOI:** 10.7759/cureus.86793

**Published:** 2025-06-26

**Authors:** Sanem Pinar Uysal, Grace Li, Benjamin R Claytor

**Affiliations:** 1 Neuromuscular Medicine, Massachusetts General and Brigham and Women’s Hospital, Boston, USA; 2 Neurology, Wright State University Boonshoft School of Medicine, Fairborn, USA; 3 Neuromuscular Medicine, Cleveland Clinic Foundation, Cleveland, USA

**Keywords:** elevated ck, glycogen storage, glycogen storage disorder, mcardle disease, metabolic myopathy, non-traumatic rhabdomyolysis, second wind phenomenon

## Abstract

Introduction

McArdle disease (glycogen storage disorder type 5) is an autosomal recessive metabolic myopathy caused by a myophosphorylase enzyme deficiency. Most patients develop symptoms during childhood; however, diagnosis is usually delayed until adulthood. Our study aimed to identify clues for an earlier recognition of this rare disease.

Materials and methods

This is a retrospective case series of 15 patients with McArdle syndrome, as diagnosed histologically and/or genetically, who were evaluated at the Cleveland Clinic Neurologic Institute. Data related to demographics, symptoms, exam findings, laboratory test results, genetic testing, muscle biopsy, and prior misdiagnoses were analyzed.

Results

Fifteen patients with McArdle disease were identified with a median age at symptom onset of 20 years (IQR=11.5-31.25). Symptoms included exertional myalgia and fatigue in all patients (n=15) and the second wind phenomenon in 53% (n=8). Creatine kinase (CK) values were elevated in 930/931 measurements (range 273-75510 IU/L), and myoglobinuria was seen in 60% (n=9). All patients exhibited significant fluctuations in CK, defined as a maximum-to-minimum ratio greater than 5 (ratio range: 5.4-276.6, median ratio: 20.4). Electromyographies (EMGs) were normal in 33% (n=5/15). Median age at diagnosis was 34 years (IQR=28-42). Median delay in diagnosis was 11 years (IQR=5-17). Ten patients (67%) were initially misdiagnosed, and the most common misdiagnosis was autoimmune myositis (n=5, 33%).

Conclusions

A lack of or underreporting of myoglobinuria or the second wind phenomenon by the patients can lead to a delay in the diagnosis of McArdle disease. Persistently elevated baseline CK without objective weakness and at times dramatically fluctuating CK levels are important diagnostic clues. Physical exam and EMG are often non-diagnostic. Increased awareness of this condition is critical to avoid misdiagnoses.

## Introduction

McArdle disease (or glycogen storage disease type 5) is an autosomal recessive metabolic myopathy caused by a deficiency of the myophosphorylase enzyme, which regulates the breakdown of glycogen to glucose-1-phosphate in skeletal muscle fibers [1]. This inherited disorder is caused by homozygous or compound heterozygous mutations in the muscle isoform glycogen phosphorylase (*PYGM*) gene located on chromosome 11q13 [2]. Recent next-generation sequencing data analyses in two cohorts of patients in the United States suggest that the disease prevalence is estimated to be between one in 7,650 and one in 42,355 [3].

The hallmark symptoms of McArdle disease are exercise intolerance, common to many metabolic myopathies, and the “second wind phenomenon,” a unique feature. The second wind phenomenon refers to an initial period of profound exercise intolerance and tachycardia, followed by an improvement in exercise capacity and a decrease in tachycardia after 6-10 minutes, due to the use of circulating free fatty acids and glucose as alternative fuels in place of glycogen [4,5]. Other clinical findings include myoglobinuria and rhabdomyolysis (defined as rapid CK elevation, usually to >10,000 U/L, as a result of muscle breakdown). Less commonly, contractures are observed, and fixed proximal weakness can develop with prolonged disease [6].

Although the onset of symptoms typically occurs during early childhood, McArdle disease is usually diagnosed in the third or fourth decade [7,8]. Most notably, nonspecific myalgias without weakness, the absence of myoglobinuria, and a lack of recognition of the second wind phenomenon may delay diagnosis until mid-adulthood. Misdiagnosis is also common in McArdle patients, affecting up to 90% due to misattributions such as growing pains or rheumatological problems [8,9].

In this study, we aim to highlight the core clinical and laboratory features that can aid in facilitating an earlier diagnosis of McArdle disease. Data included in this study have been previously presented as posters at the American Association of Neuromuscular and Electrodiagnostic Medicine Annual Meeting, September 23, 2022, and November 3, 2023.

## Materials and methods

This is a retrospective study of patients with histologically or genetically proven McArdle disease evaluated at the Cleveland Clinic Neurologic Institute between 2000 and 2022. Inclusion criteria were either a positive muscle biopsy demonstrating absence of staining for myophosphorylase or positive genetic testing demonstrating two pathogenic or likely pathogenic mutations in the *PYGM* gene. Mutations classified as pathogenic met the criteria outlined by the American College of Medical Genetics and Genomics (ACMG), and likely pathogenic mutations were determined based on a combination of in silico analysis, consideration of population frequencies of a variant, and reports of identified variants in other individuals with McArdle disease. Segregation studies were conducted at the discretion of the ordering provider. Data related to demographics, comorbidities, EMG findings, onset and type of symptoms, exam findings, pertinent family history, CK and renal function test results, genetic testing, muscle biopsy, date of diagnosis, prior misdiagnoses, clinical course, and physical activity level were collected retrospectively from medical chart review. The timing and frequency of CK measurements were at the discretion of the ordering provider, and the decisions for specific stains utilized for the muscle biopsies were at the discretion of the interpreting pathologist.

Descriptive statistics were summarized by mean and/or median with interquartile range (IQR) for continuous variables and count with percentage for categorical variables. The study was approved by the Cleveland Clinic Institutional Review Board (study number 22-978), and participant consent was waived.

## Results

The study included 15 patients, 60% of whom were male. All patients were Caucasian. Median follow-up, as defined by time from initial visit in our system for muscle-specific symptoms to the last evaluation, was 25 months (IQR=6-130). Of the twelve patients who had a clearly defined age of onset, the median age at first symptom was 20 years (IQR=11.5-31.25), ranging from six months to 69 years. Three patients did not have a documented age of onset, but two of them reported symptom onset during early childhood. Age of onset was before 10 years in 36% (n=5), 10-29 years in 21% (n=3), 20-29 years in 21% (n=3), 30-39 years in 7% (n=1) and after 50 years in 14% (n=2) (Table 1).

**Table 1:** Demographic and clinical features of the study population The total N available represents the total number of patients for whom data points were available for each specific variable.

	N	Total N available
Male sex (n, %)	9 (60%)	15
Caucasian ethnicity (n, %)	15 (100%)	15
Follow up time (median (Q1, Q3), months)	25 (6-130)	15
Age at symptom onset (median (Q1, Q3), years)	20 (11.5-31.25)	14
Before the age of 10 years (n, %)	5 (36%)	14
Between 10 and 19 years (n, %)	3 (21%)	14
Between 20 and 29 years (n, %)	3 (21%)	14
Between 30 and 39 years (n, %)	1 (7.1%)	14
Between 40 and 49 years (n, %)	0	14
After the age of 50 years (n, %)	2 (14%)	14
Positive family history (n, %)	3 (20%)	15
Age at diagnosis (median (Q1, Q3), years)	34 (28-42)	15
Misdiagnoses (n, %)	10 (67%)	15
Polymyositis/dermatomyositis (n, %)	5 (50%)	10
Mitochondrial myopathy (n, %)	2 (20%)	10
Symptoms		
Myalgia (n, %)	15 (100%)	15
Fatigue (n, %)	15 (100%)	15
Rhabdomyolysis (n, %)	14 (97%)	15
Myoglobinuria (n, %)	9 (60%)	15
Second wind phenomenon (n, %)	8 (53%)	15
Clinical course		
Worsening symptoms	10 (67%)	15
Ambulatory at last visit	15 (100%)	15

Three patients (20%) had a family history of McArdle disease. Two patients included in the study were siblings, and the older sibling was diagnosed at age six in the context of elevated CK, muscle pain, and motor delays. This led to CK measurement in the younger sibling, which was elevated, and family variant testing revealed identical compound heterozygote *PYGM* mutations. One patient had two siblings with elevated CK and muscle pain, and the first McArdle diagnosis in the family was made in one of the siblings based on a positive muscle biopsy; however, no additional records were available. There was no known consanguinity in either of the affected families. No documented cascade genetic testing was performed in any of the patients' families in this study. Two patients (13%) had family members with undiagnosed possible neuromuscular diseases that were not well characterized.

All patients initially presented with exertional myalgia and fatigue. Other symptoms included rhabdomyolysis in 93% (n=14), myoglobinuria in 60% (n=9), and second wind phenomenon in 53% (n=8) (Table 1). Notably, the second wind phenomenon was recognized by the patient in 47% (n=7) and by the medical provider in 53% (n=8) of cases, with all mutual identifications except for one patient, who was identified by the provider alone. Physical exams revealed mild proximal weakness in 60% (n=9) and were normal in the remaining patients.

The diagnosis was confirmed by genetic testing alone in 53% of cases (n=8), while in 40% of cases (n=6), the diagnosis was confirmed exclusively via muscle biopsy based on the absence of myophosphorylase staining. Only one patient underwent both muscle biopsy and genetic testing (Table 2). Two additional patients underwent muscle biopsy at an outside institution, and these were not diagnostic of McArdle disease; however, immunohistochemical staining for myophosphorylase was not reported to have been performed. In the nine subjects who underwent genetic testing, five received a diagnosis from a next-generation sequencing multi-gene panel, two via whole-exome sequencing, one via familial variant testing, and one subject had one pathogenic variant identified on a next-generation sequencing panel and then subsequently had a second likely pathogenic variant identified via Sanger sequencing of the *PYGM* gene. Not surprisingly, the most common mutation identified was p.Arg50, which produces a premature stop codon and is the most common variant in cohort studies [3]. Of the seven patients with compound heterozygote mutations, three underwent segregation analysis (Table 2). All the missense mutations identified in this study have been previously reported in patients with McArdle disease, except for c.2443G>A (p.Asp815Asn), which in silico analysis predicted would not be tolerated. One variant in our study, p.Val238Cys fs*15, is expected to lead to a frameshift and a premature stop codon, resulting in a classification as pathogenic by the ACMG; however, this variant has not been previously reported in a McArdle disease patient.

**Table 2:** Diagnostic findings of the study population

	N	Total N available
Physical exam findings		
Mild proximal weakness (n, %)	9 (60%)	15
Normal (n, %)	6 (40%)	15
Lab findings		
Abnormal creatine kinase at any point (>199 U/L) (n, %)	930 (99%)	931
CK max/min ratio >5	15 (100%)	15
Abnormal creatinine on recent testing (>1.2 mg/dL) (n, %)	0	13
Abnormal EMG findings (n, %)	6 (40%)	11
Genetic testing findings		
Compound heterozygote c.148C>T (p.Arg50) and c.613G>A (p.Gly205Ser), confirmed segregation analysis	2 (22%)	9
Compound heterozygote c.148C>T (p.Arg50) and c.1466C>G (p.Pro489Arg)	1 (11%)	9
Compound heterozygote c.148C>T(p.Arg50) and c.711dupT (p.Val238Cys fs*15)	1 (11%)	9
Compound heterozygote c.148C>T(p.Arg50)and c.2443G>A (p.Asp815Asn)	1 (11%)	9
Compound heterozygote c.613G>A (p.Gly205Ser) and c.1628A>C (p.Lys543Thr)	1 (11%)	9
Homozygous c.148C>T (p.Arg50)	2 (22%)	9
Compound heterozygote c.148C>T(p.Arg50) and c.1190T>C (p.Leu397Pro), confirmed segregation analysis	1 (11%)	9
Positive muscle biopsy	7 (78%)	9

A neurologist or neuromuscular specialist diagnosed all cases during their evaluation. The median age at diagnosis was 34 years (IQR=28-42). Duration of diagnostic delay ranged from 1 to 30 years (median=11, IQR=5-17).

**Figure 1:**
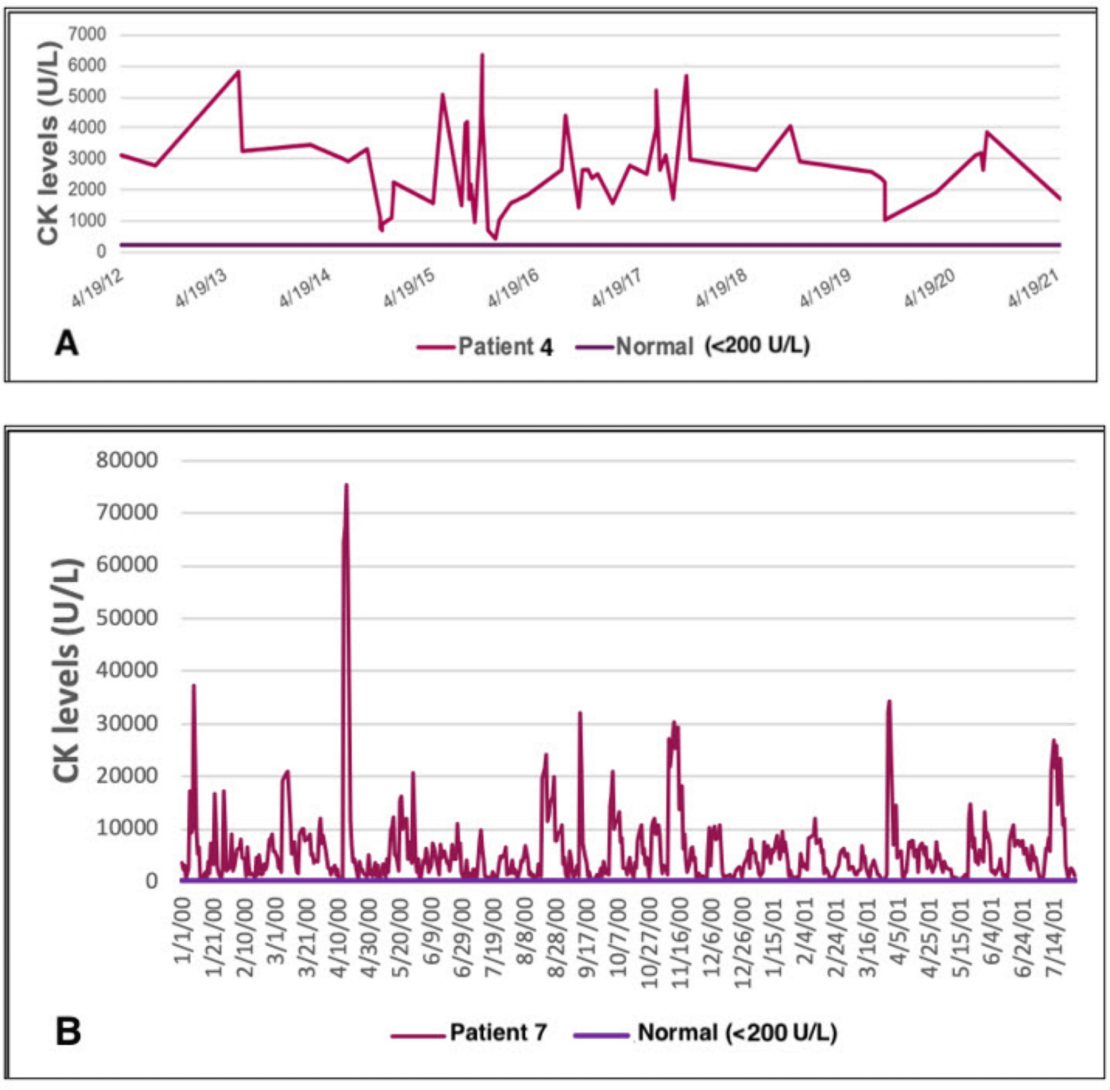
Stereotypical CK fluctuations over time, CK max/min >5 (A) CK fluctuation for patient ID4 over nine years. (B) CK fluctuation for patient ID7 over 1.5 years. CK: creatine kinase

CK levels were measured a total of 931 times (median=27, IQR=5-31). Among 931 measurements, CK was normal only once. CK values varied from 273 to 75510 U/L, with maximums above 3000 U/L in all but one patient. 809/931 CK measurements (86%) were above 1000 U/L, and all patients had CK elevation above 1000 at least once. Significant CK fluctuations, defined as max/min ratio >5, were observed in all patients (median=20.4, IQR=6.8-42.1). Typical CK fluctuations over time are demonstrated for two patients in Figure 1 who underwent numerous CK measurements over several years. Creatinine was within normal limits for all patients with available results.

EMGs were normal in 33% (n=5) and showed mild non-diagnostic abnormalities in 40% of patients (n=6) (Table 2). These non-diagnostic abnormalities included myopathic-appearing units in a restricted number of muscles (1-2), fibrillation potentials in one or two muscles in isolation, or findings unrelated to the muscle disease, such as a sensorimotor polyneuropathy. Three patients had their EMG performed at an outside institution, so the results were based on the available reports. For the eight patients who underwent EMG at our institution, five had a myopathy protocolled study with one upper and lower extremity sensory and motor nerve conduction study (NCS) and needle examination of five lower extremity muscles, three proximal to the knee, and six upper extremity muscles, four proximal to the elbow, and a cervical paraspinal muscle. One patient had an isolated lower extremity study consisting of two sensory and two motor NCS and five muscles sampled on needle examination, and one patient had an upper extremity-only study composed of three sensory and two motor NCS and seven muscles sampled on needle examination. The last patient had separate upper and lower extremity studies. Four patients did not undergo EMG testing. Of the patients with a normal strength exam (n=6), five had normal EMGs, while one had nonspecific fibrillation potentials in the left gastrocnemius and left biceps muscles.

Ten patients (67%) were misdiagnosed, the most common diagnosis being polymyositis or dermatomyositis in five patients (33%). Of the patients misdiagnosed with an inflammatory myopathy, one had a weakly positive anti-nuclear antibody, but none of the others harbored any myositis-specific antibodies. Additionally, three of the five patients underwent muscle biopsy, which was negative for any auto-inflammatory findings in all cases. It appears that the diagnosis of an inflammatory myopathy was based solely on the presence of muscle pain and elevated CK. Other misdiagnoses included mitochondrial myopathy (n=1), which was based on a muscle biopsy with rare COX-negative fibers when the subject was 71 years old, and glycogen storage disease type 3 (n=1), which was based on a historic muscle biopsy that showed reduced staining of glycogen debranching enzyme. Other misdiagnoses, including statin myopathy (n=1), malignant hyperthermia (n=1), and nonspecific myopathy (n=1), were also based on clinical impression alone (Table 1). Three patients who were misdiagnosed were treated with immunotherapy for the presumed conditions, including intravenous immune globulin (IVIG), prednisone, methotrexate, and azathioprine. One patient had documented side effects from IVIG, including headache and rash. Another patient had documented side effects from prednisone, including rapid weight gain and abdominal striae.

## Discussion

In this study, we examined the clinical characteristics of a group of McArdle disease patients evaluated at our institution. We aimed to identify clinical features to facilitate an early diagnosis. The most common age for symptom onset was before 10 years, and most patients developed initial symptoms prior to 20 years of age. This is consistent with other cohorts of McArdle disease patients, who typically experience symptom onset in childhood [1,10,11]. The mean age at diagnosis was 34 years for our patients, which is also consistent with prior studies [7,8,12,13].

It is notable, however, that two patients in our study developed symptoms after the age of 50, one of whom had no documented muscle-specific symptoms until the age of 68, when they presented with symptoms of progressive weakness and exercise intolerance. The other patient developed muscle pain at the age of 52 when they were prescribed a statin, and the only other possible McArdle-related symptom documented prior to this was difficulty with long-distance running. This highlights the need to consider a McArdle diagnosis in older adults with exercise intolerance or muscle pain who have elevated and fluctuating CK levels, even in the absence of symptoms during childhood.

In this study, there was a wide range in the duration of diagnostic delay, from one to 30 years (median=11, IQR=5-17), and long diagnostic delays have also been demonstrated in other studies [9,12]. All patients were ultimately diagnosed during evaluations by their neurologists or neuromuscular specialists, indicating the need for timely referral to neurology. More than half of our patients received their diagnosis more than a decade ago, which may explain the higher utilization of muscle biopsy for diagnostic confirmation.

All our patients initially presented with non-specific complaints of fatigue, subjective weakness, and myalgia induced by exercise. The second wind phenomenon, a distinguishing characteristic of McArdle disease, was recognized by slightly over half of the study population, suggesting that this clinical symptom has limited diagnostic sensitivity. A higher proportion of patients (86%) with an identified second wind phenomenon during their lifetime has been reported in the literature [12]. These findings, in combination, highlight the importance of screening for this symptom during the initial evaluation of patients presenting with exercise intolerance. The second wind phenomenon is likely still underrecognized, especially if the symptom onset was during childhood, so the absence of a second wind should not dissuade consideration of a McArdle disease diagnosis.

All patients in our study population had abnormal CK levels, with prominent fluctuation in CK, characterized by a maximum-to-minimum ratio greater than 5. Similarly, in a Spanish study, basal hyperCKemia, defined as a CK level above 200 U/L, was present in 99% of patients, with 79% having a level above 1000 [12]. It is important, however, that CK levels be interpreted in the context of the patient's ethnicity, as population-based studies have demonstrated that Black individuals have a higher basal CK level [14]. Notably, more than fivefold elevations or reductions from the immediately previous CK values occurred in all our patients and were not associated with clinical deterioration. This rapid and dynamic CK fluctuation in the absence of significant weakness or EMG abnormalities can serve as an important clue to the diagnosis. Additionally, despite at times very high CK levels of >10,000, none of the patients in our cohort had evidence of renal dysfunction.

In contrast to CK levels, our study demonstrated that clinical examination and electrodiagnostic findings are minor and non-diagnostic when present, similar to the data reported from the European registry of McArdle patients. However, fixed proximal weakness has been described in older patients [9]. The clinical mismatch between relatively benign exams and electrodiagnostic testing results and CK levels, as well as fluctuating CKemia, is a useful clue to the diagnosis of McArdle disease. While CK levels can be elevated in other glycogen storage diseases, including glycogen storage disease type 3, very high levels of CK (>5000) are more typical of McArdle disease [15].

Unfortunately, misdiagnosis continues to play an important role in delaying the diagnosis of McArdle disease. The majority of our patients received at least one misdiagnosis prior to confirmation of McArdle disease. Additionally, 30% of these patients received inappropriate immunotherapy. Misdiagnoses and the delay in McArdle diagnosis are likely multifactorial, due to the rarity of this disease, heterogeneity in the severity of signs and symptoms, and the broad differential for exercise intolerance.

This study has several strengths, including the availability of detailed clinical data and longitudinal follow-up for patients in neurology or neuromuscular clinics. Limitations include the study's retrospective nature, the inclusion of patients from a single institution, the small number of patients due to the rarity of the disease, and the potential for incomplete data. Many patients were evaluated at other institutions first; therefore, the nature and extent of these prior evaluations, including the number and type of physicians they consulted before receiving a correct diagnosis, were not known. The rarity of McArdle disease limits the sample size and the ability to conduct statistical analyses beyond descriptive ones.

## Conclusions

A lack of recognition or underreporting of myoglobinuria or the second wind phenomenon by patients may delay the diagnosis of McArdle disease. These can be helpful signs if present, but their absence should not exclude consideration of a McArdle disease diagnosis. Persistent and significantly elevated CK without normalization in the absence of objective weakness or electrodiagnostic abnormalities is an important diagnostic key. Significant fluctuations in CK levels, with a maximum-to-minimum ratio greater than 5, without a clear clinical association, are another diagnostic clue. Improving awareness of McArdle disease is crucial for preventing misdiagnoses and unnecessary treatments, as well as facilitating early diagnosis and prompt, effective management. In the current era of widely available genetic testing, patients with possible symptoms of McArdle disease, including muscle pain and exercise intolerance, who also have persistently elevated and fluctuating CK levels, should undergo genetic evaluation to help facilitate early diagnosis. Screening of a patient’s first-degree relatives for muscle-specific symptoms or elevated CK, along with the help of genetic counselors, may also help facilitate earlier diagnoses in affected family members.
